# Periodontitis and Intra-Oral Plasmacytosis in a Noonan's Syndrome Patient

**DOI:** 10.1155/2022/1379769

**Published:** 2022-12-26

**Authors:** Hadeel Alqatami, Khalid N. Said, Adham A. Ammar, Hani Nazzal

**Affiliations:** ^1^Pediatric Dental Department, Hamad Dental Centre, Doha, Qatar; ^2^Department of Dentistry, Oral Health Institute, Al Wakra Hospital, Hamad Medical Corporation, Doha, Qatar; ^3^College of Dental Medicine, Qatar University, Doha, Qatar; ^4^Surrey and Sussex Health care Trust, Surrey, UK

## Abstract

A 13-year-old female patient with Noonan's syndrome, intra-oral periodontitis, and associated periodontal lesions is presented in this case report. The patient suffered early onset severe molar pattern periodontitis and recurrent intra-oral inflammatory lesions, pyogenic granuloma, and plasmacytosis, which were excised and controlled using a strict oral hygiene protocol based on long-term use of chlorhexidine-based products as auxiliary aid to regular home care and brushing.

## 1. Introduction

Noonan syndrome (NS) is an autosomal dominant disorder characterized by a spectrum of congenital heart defects, webbed neck, unusual chest deformity, mild mental deficiencies, short stature, facial deformities, and bleeding tendencies [1] (OMIM #163950).

Several oral manifestations have been associated with this syndrome including high arched palate, malocclusion, micrognathia, numerical tooth anomalies, mandibular cysts resembling cherubism, extensive caries, periodontal diseases, and giant cell lesions [1, 2].

The following case describes the dental and periodontal management of a 13-year-old female patient with a combination of severe periodontitis in localized molar pattern, pyogenic granuloma, and plasmacytosis lesions.

## 2. Case Report

The patient was referred by her cardiologist to the Pediatric Dental Department at Hamad Medical Corporation, Doha, Qatar, for the management of gingival bleeding. Medically, the patient presented in with NS heterozygous *SOS1*, *MTO1*, and *MYPNS* genes, hypertrophic cardiomyopathy with myomectomy and mitral valve repair in 2017, short stature, scoliosis with congenital tethering of spinal cord, and transient synovitis. The patient was prescribed *β*1 blocker, aspirin, Lasix, iron supplement, and growth hormone (Norditropin®).

**Figure 1:**
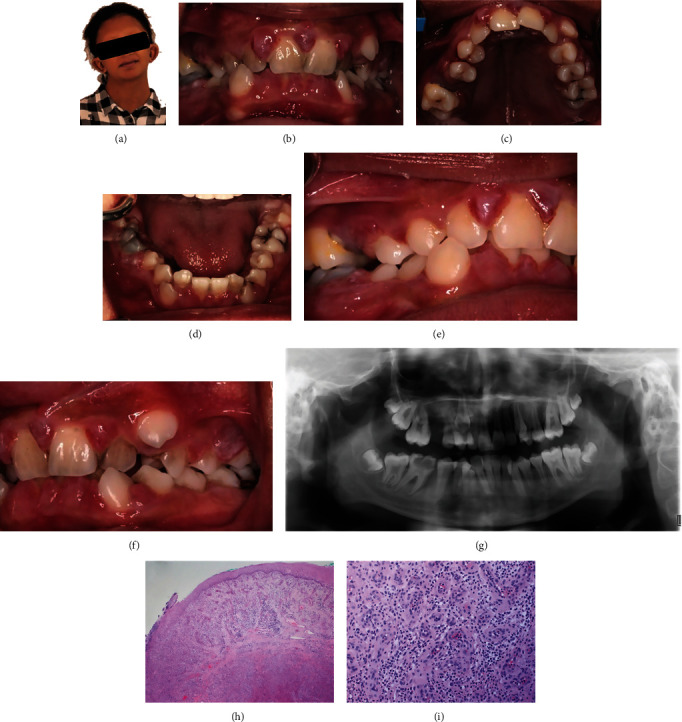
(a) Extra-oral frontal view at presentation. (b)–(f) Intra-oral views at presentation. (g) Orthopantomogram at presentation. (h) and (i) Hematoxylin & eosin stained images of first excisional biopsy of gingival lesion palatal to teeth #8 and #9. (h) Photomicrograph depicting surface squamous mucosa exhibiting focal ulceration, and underlying submucosal proliferation of capillary sized blood vessels with lobular arrangement (×40). (i) High power view showing proliferation of capillaries admixed with numerous mononuclear and polymorphonuclear inflammatory cells (×200).

Dentally, the patient presented with high cariogenic diet, which involved regular consumption of PediaSure® dietary supplements and high plaque accumulation on her teeth secondary to poor oral hygiene practice. Periodontally, the patient presented with edematous gingivae with erythema and enlargement associated with bleeding on probing (Figure 1), localized severe angular periodontal bone loss mesial to teeth #3 and #14 associated with deep probing depth of 5 mm, bleeding on probing, furcation involvement, and severe clinical attachment loss. A class 2 mobility was observed with tooth #3, whereas a moderate bone loss was observed interproximally between teeth #13 and #14.

In addition, the patient presented with localized, soft, and bluish red soft tissue enlargement interproximally (mainly palatally) between teeth #7, #8, and #9. Other clinical findings include an impacted tooth #6 with an associated supplemental premolar tooth, space loss, crowding in both arches, high arched palate, micrognathia, and taurodontism of teeth #2 and #15. Clinical and radiographic findings are summarized in Figure 1.

**Figure 2:**
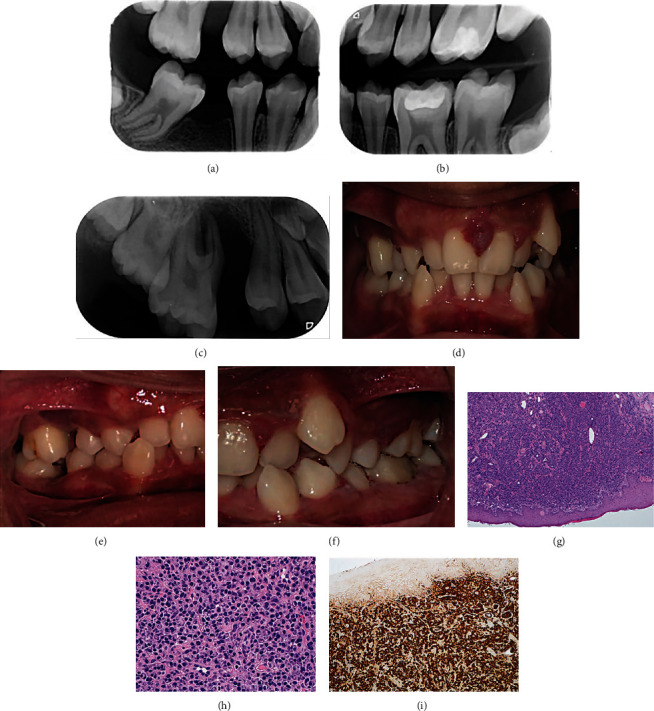
(a)–(c) Follow-up radiographs at 6 months. (d)–(h) Intra-oral views at 6 months follow-up. (h) and (i) Hematoxylin & eosin stained images of second excisional biopsy of interdental gingival lesion to teeth #8 and #9 showing. (i) Hyperplastic squamous mucosa with underlying nodular sheet-like plasma cells in a background of collagenized stroma composed of fibroblasts and myofibroblasts. (j) High power view showing morphologically monotonous and diffused cells with no mitotic figures or nuclear atypia indicative of plasma cell infiltrate. (k) Immunostains for kappa and lambda showing a strong diffused positivity with no light chain restriction. Findings were indicative of polyclonality of the lesion.

Communication with the patient's physician ruled out an associated hemorrhagic tendency, whereas antibiotic prophylaxis against infective endocarditis was recommended and carried out during all future invasive treatments. An interdisciplinary treatment plan was developed and performed (Figure 2), whereby the patient underwent an intensive home preventive care, regular scaling with selective root planning of deep periodontal pockets, restoration of the carious teeth #14 and #19, and extraction of the unrestorable tooth #30. An excisional biopsy of the interdental soft tissue masses at the maxillary incisor region was performed. The histopathological assessment showed proliferation of capillaries admixed with numerous mononuclear and polymorphonuclear inflammatory cells consistent with pyogenic granuloma (Figure 1). Orthodontic intervention was delayed until the periodontal disease status is controlled.

Up to 6 months recall, the initial treatment plan was found to be partially successful in improving the patient's oral health with initial resolution of gingival edema and erythema, and the periodontal and gingival lesions associated with tooth #3 (Figure 2). In addition, the interdental masses adjacent to teeth #8 and #9 had recurred.

A second treatment phase included extraction of tooth #3 as a result of the severe bone loss, severe mobility, and possible associated periodontic–endodontic lesion. In addition, a second excisional biopsy of the recurrent lesions between teeth #8 and #9 along with a labial frenoplasty, which aimed at releasing the aberrant low attachment maxillary labial frenum thought to contribute to the recurrent lesion, was performed. The second histopathological assessment showed evidence of hyperplastic squamous mucosa with underlying nodular sheet-like plasma cells in a background of collagenized stroma composed of fibroblasts and myofibroblasts, and morphologically monotonous and diffused cells with no mitotic figures or nuclear atypia indicative of plasma cell infiltrate (Figure 2). In addition, immunostains for kappa and lambda showed a strong diffused positivity with no light chain restriction (Figure 2). The histopathological findings, therefore, were indicative of intra-oral plasmacytosis.

**Figure 3:**
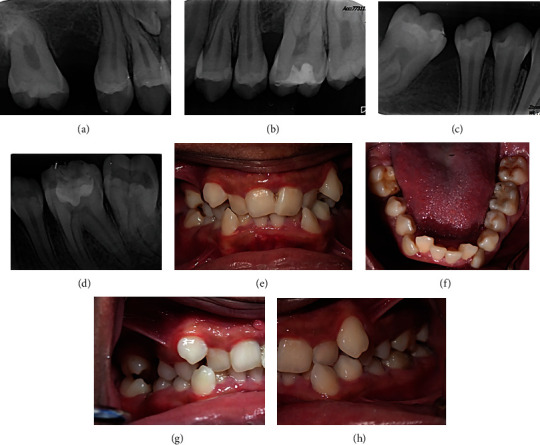
Periapical radiographs and intra-oral images taken at 6 months recall from second excisional biopsy (no recurrence was observed up to 12 months).

The patient's home care plan was modified by introducing twice daily tooth brushing using chlorhexidine 0.12% toothpaste and the application of chlorhexidine 0.2% spray to the site of recurrent lesions before bedtime and in the morning, daily, during the healing process. The patient was maintained on the chlorhexidine 0.12% toothpaste thereafter. This approach led to total resolution of inflammation signs and prevented the recurrence of the lesion between teeth #8 and #9 up to 12 months post-operatively (Figure 3).

## 3. Discussion

Although the patient presented with several characteristics in line with NS, this case report focuses on the diagnosis and management of the gingival masses and periodontitis lesions in patients with NS.

The incidence of periodontitis in children is low and usually is linked to the presence of a systemic diseases, such as severe systemic immune alterations or associated with hereditary disorders, such as Papillon–Lefèvre syndrome [3]. Among adolescents and young adults, the incidence of molar-incisor pattern of periodontitis, historically known as localized juvenile periodontitis, remains low at less than 0.5% [4]. Nevertheless, the devastating health, nutrition, and psychological effects, associated with pathologic migration of teeth and early loss of dentition, are high.

The patient presented with a combination of localized molar pattern of severe periodontitis, which is generally rare in children and infrequently reported in patients with NS [1, 5, 6]. Torres-Carmona et al. [5] reported the presence of severe periodontitis in a 15- and 18-year-old NS patients with molar-incisor pattern of alveolar bone destruction. Similar findings were observed in this case, where severe periodontitis was associated with advanced bone loss and clinical attachment loss mesial to teeth #3, #14, and #30.

The patient also presented with two distinct gingival masses that showed histopathological presentations of pyogenic granuloma and intra-oral plasmacytosis.

Pyogenic granuloma is considered a reactive tumor like lesion developed in response to different stimuli, such as low grade local irritation, trauma, hormone changes, and certain drugs [7]. This condition is prevalent in young adult females as a possible result of the vascular effects of female hormonal changes [7]. Oral lesions usually involve the anterior maxillary interdental gingiva with predilection to affect the facial aspects. The poor oral hygiene with associated supra and subgingival plaque and calculus coupled with hormonal changes might have contributed to the development of these lesions in this patient.

Mucous membrane plasmacytosis is a rare, benign disorder where plasma cells infiltrate the mucous membranes including the buccal mucosa, palate, gingiva, lips, and tongue [8]. Different terms have been used to describe this condition, such as atypical/idiopathic/allergic gingivostomatitis, plasmacytosis circumorificialis, plasmacytosis mucosae, plasma cell-gingivitis, plasmacytosis of the gingival, plasma-cell cheilitis, and plasma-cell orificial mucositis [8]. Currently, variants of the term plasma-cell orificial mucositis, such as mucous membrane plasmacytosis and plasma-cell mucositis, have been used in the majority of cases [8]. Different treatments of this condition, such as corticosteroids, antibiotics, liquid nitrogen, CO_2_ laser, electrocoagulation, excision of the tissue, and radiation therapy, have been reported in the literature [8]. None of these treatment modalities has been shown to be more superior [8].

In the presented case, a combination of good home care supplemented with chlorhexidine toothpaste and spray, regular scaling/root debridement, and extraction of the teeth with questionable long-term prognosis were successful in controlling the periodontal condition, whereas surgical excision and long-term use of chlorhexidine toothpaste/spray appeared effective in resolving the gingival pathological lesions for 12 months. Patients with NS present with multiple physical disabilities and some with mental complications that may limit their ability to effectively maintain oral health; therefore, the use of additional oral hygiene adjuncts is helpful in improving their oral hygiene.

Careful review of the patient especially with regard to the recurrence of the intra-oral plasmacytosis is essential. Despite the good prognosis associated with intra-oral plasmacytosis, few studies have demonstrated progression into the larynx occasionally causing airway obstruction [8].

Interestingly, the patient has mutations in *SOS1* gene, which has been linked to the development of gingival fibromatosis, which is a rare hereditary disease characterized by progressive slow enlargement of interdental and attached gingiva [9]. Although not observed in this case, further investigations are needed to understand the possible correlation between the *SOS1* gene and the tendency to develop mucosal lesions, such as gingival plasmacytosis.

On reflection, microbiological sampling of the infected periodontal pockets in order to assess the microbial flora associated with the severe periodontal tissue destruction could have helped in the management of any persistent lesions in the future.

This case, in addition to previously published reports on NS, highlights the risk of severe periodontitis in young age, leading to premature loss of molar teeth, in addition to the high inflammatory systemic burden associated with periodontitis. Given the nature of the syndrome, most patients suffer from malnutrition, which is worsened by presence of intra-oral swelling and infections. Therefore, careful gingival, periodontal, and soft tissue assessments and frequent recalls in the hygiene or periodontal clinics are recommended in such patients. Long-term use of chlorhexidine toothpaste and other preparations may play a role in prevention of recurrence of intra-oral plasmacytosis. However, further studies are required to confirm this observation.

## Data Availability

Data supporting this research article are available from the corresponding author or first author on reasonable request.

## References

[1] Sugar A. W., Ezsias A., Bloom A. L., Morcos W. E. (1994). Orthognathic surgery in a patient with Noonan’s syndrome. *Journal of Oral and Maxillofacial Surgery*.

[2] Hwang I., Lee Y., Sim D., Mah Y. (2018). Oral features in a child with Noonan syndrome: a case report. *Journal of the Korean Academy of Pediatric Dentistry*.

[3] Meyle J., Gonzáles J. R. (2000). Influences of systemic diseases on periodontitis in children and adolescents. *Periodontology 2000*.

[4] Nassar M. M., Afifi O., Deprez R. D. (1994). The prevalence of localized juvenile periodontitis in Saudi subjects. *Journal of Periodontology*.

[5] Torres-Carmona M. A., Arenas-Sordo M. L., Saavedra-Ontiveros D., Sánchez-Guerrero M. C. (1991). Periodontal disease in Noonan’s syndrome. *Boletín Médico del Hospital Infantil de México*.

[6] Ortega Ade O., Guaré Rde O., Kawaji N. S., Ciamponi A. L. (2008). Orofacial aspects in Noonan syndrome: 2 case report. *Journal of Dentistry for Children*.

[7] Jafarzadeh H., Sanatkhani M., Mohtasham N. (2006). Oral pyogenic granuloma: a review. *Journal of Oral Science*.

[8] Bharti R., Smith D. R. (2003). Mucous membrane plasmacytosis: a case report and review of the literature. *Dermatology Online Journal*.

[9] Gawron K., Łazarz-Bartyzel K., Potempa J., Chomyszyn-Gajewska M. (2016). Gingival fibromatosis: clinical, molecular and therapeutic issues. *Orphanet Journal of Rare Diseases*.

